# Medium- to Long-Term Outcomes after Reverse Total Shoulder Arthroplasty with a Standard Long Stem

**DOI:** 10.3390/jcm11092274

**Published:** 2022-04-19

**Authors:** Matthias Bülhoff, Felix Zeifang, Caroline Welters, Tobias Renkawitz, Marcus Schiltenwolf, Anna-K. Tross

**Affiliations:** 1 Clinic for Orthopaedic Surgery, Heidelberg University Hospital, Schlierbacher Landstraße 200a, 69118 Heidelberg, Germany; matthias.buelhoff@med.uni-heidelberg.de (M.B.); tobias.renkawitz@med.uni-heidelberg.de (T.R.); marcus.schiltenwolf@med.uni-heidelberg.de (M.S.); 2 Ethianum Clinic Heidelberg, Voßstraße 6, 69115 Heidelberg, Germany; felix.zeifang@ethianum.de; 3 Clinic for Dermatology, Munich Municipal Hospital Group, Thalkirchnerstrasse 48, 80337 Munich, Germany; c.welters@hotmail.de

**Keywords:** shoulder, reverse arthroplasty, polyethylene wear, humeral loosening, glenoid loosening, scapular notching

## Abstract

Background: Long-term clinical and radiographic outcome data after standard cemented long-stem reverse shoulder arthroplasty (RSA) remain underreported. The aim of this study is to report on medium- to long-term data of patients over 60 years of age. Methods: The same type of RSA (Aequalis Reverse II, Memphis, TN, USA) was implanted in 27 patients with a mean age of 73 years (range 61–84). Indications for RSA were cuff tear arthropathy (CTA) in 25 cases and osteoarthritis (OA) in two cases. Pre- and postoperative Constant Score was assessed and component loosening, polyethylene wear, scapular notching and revision rates were recorded at a mean clinical follow-up (FU) of 127.6 months (SD ± 33.7; range 83–185). Results: The mean-adjusted CS (aCS) improved from 30.0 (range 10–59) to 95.0 (range 33–141) points (*p* < 0.001). Glenoid loosening was found in two (9.1%) and stem loosening was found in three (13.6%) cases. Polyethylene wear was observed in four (18.2%) cases. Scapular notching appeared in 15 (68.2%) cases but was not associated with poor aCS (*p* = 0.423), high levels of pain (*p* = 0.798) or external rotation (*p* = 0.229). Revision surgery was necessary in three (11.1%) cases. Conclusions: RSA with a cemented standard long stem leads to improvement in forward elevation, abduction and pain after a mean FU of 10 years. However, external rotation does not improve with this prosthetic design. Moreover, scapular notching is observed in the majority of cases, and revision rates (11.1%) as well as humeral loosening rates (13.6%) remain a concern. Level of evidence: Level 4, retrospective cohort study.

## 1. Introduction

Within the past three decades, reverse shoulder arthroplasty (RSA) has been established as a valuable surgical treatment option for end-stage degenerative pathologies of the shoulder joint [1]. However, in comparison to other joint replacements [2], RSA must still be considered a rather new technique; and in some countries, such as the United States, RSAs were not implanted before the Food and Drug Administration approved them in 2003 [3].

The original Grammont Delta-III-prosthesis (DePuy), as the prototype of all modern reverse shoulder arthroplasty designs [4], was launched in 1991 for the treatment of cuff tear arthropathy (CTA) with painful pseudoparalysis [5]. Through medialization and distalization of the center of rotation of the shoulder joint, the delta muscle becomes a compensator for the deficient rotator cuff muscles in RSA [6].

Today, modern RSA designs are derived from the original Grammont Delta-III-prosthesis and are implanted for a wider range of indications such as primary osteoarthritis (OA), massive rotator cuff tears, post-traumatic OA, irreparable proximal humeral fractures, rheumatoid arthritis, in tumor situations and as a revision option after anatomic hemi or total shoulder replacement [5,7,8]. As indications for RSA expand, the absolute numbers of implanted RSAs have recently surpassed the numbers of anatomic shoulder replacements [9].

Treatment with RSA has been shown to result in decreased pain levels and restored function [10,11]. Revision rates range between 0% and 13%, with aseptic component loosening on the glenoid side being one of the main reasons for revision surgery [6,12,13,14]. Complications of the humeral stem include subsidence, aseptic loosening and unscrewing at the humeral stem–neck interface and can become a major reason for revision surgery over time [15,16]. A unique radiographic phenomenon after RSA remains scapular notching, which is defined as scapular neck erosion caused by repetitive mechanical contact of the humeral component with the scapular neck [17]. Associations between scapular notching and clinical outcome [18], glenoid loosening rates [10] and time of follow-up [12] have been demonstrated in the past; however, the true impact remains a controversial topic [19,20].

The aim of this study was to report on medium- to long-term functional results, satisfaction, survival rates and radiographic changes after the implantation of a traditional long-stem reverse prosthesis for patients with cuff tear arthropathy and primary osteoarthritis over the age of 60. We hypothesized that clinical outcome parameters would still improve at minimum follow-up of 5 years.

## 2. Materials and Methods

**Figure 1:**
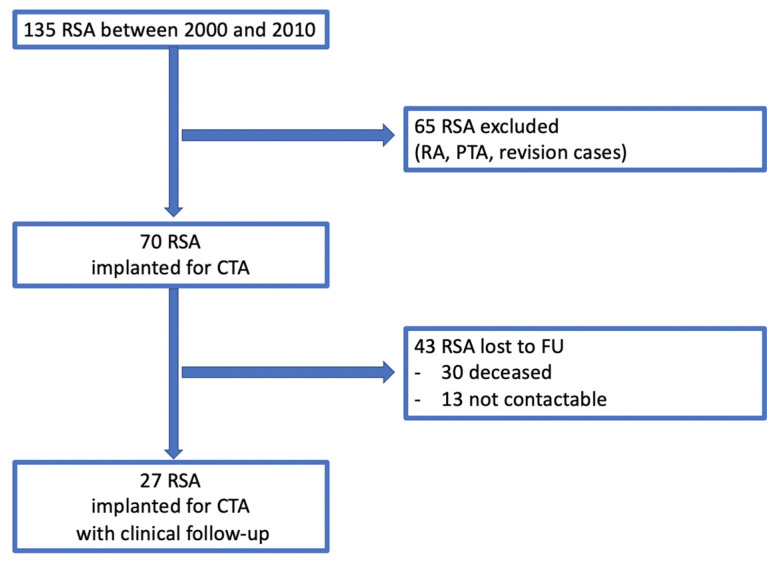
Flow chart. RSA = reverse shoulder arthroplasty; RA = rheumatoid arthritis; PTA = post traumatic arthritis; CTA = cuff tear arthropathy; FU = follow-up.

A retrospective analysis of prospectively enrolled data of the institution’s database was performed. In total, RSA was performed in 135 cases for various indications between January 2000 and December 2010. Local ethics committee approval was obtained prior to the start of the study (# S-305/2007). Surgeries were performed in a single surgery center by or under the supervision of two experienced orthopaedic surgeons. Inclusion criteria were (1) CTA (2) primary OA, (3) minimum follow-up (FU) of five years and (4) primary treatment with the same type of standard long-stem (standard long stem has been defined before as a stem length of approximately 100 mm [21]) reverse prosthesis (Aequalis Reverse II, Memphis, TN, USA) and (5) written consent with the study. Exclusion criteria were arthroplasty prior to the index surgery, rheumatoid arthritis and fractures. According to the exclusion criteria, 65 cases were excluded (a flowchart is provided with Figure 1).

A total of 65 RSA were excluded according to the exclusion criteria (RA = rheumatoid arthritis; PTA = post-traumatic arthritis). A total of 43 RSA were lost to FU, leaving 27 RSA with clinical FU.

The overall cohort included in this study comprised 27 patients (23 females (85%), 4 males (15%) with a mean clinical FU of 127.6 (SD ± 33.7; range, 83–185) months and a mean radiographic FU of 126.4 (SD ± 34.3; range 83–185) months. Patients were invited for clinical and radiographic assessment between December 2016 and September 2017. Indications for RSA were CTA in 25 cases (92.6%) and primary OA in two cases (7.4%). Patients had a mean age of 72.6 (SD ± 5.4; range 61–84) years at the time of implantation, the right shoulder was treated in 23 (85%) cases and the left in 4 (15%) cases. The dominant shoulder was treated in 18 cases (67%). Mean operation time was 98 min (SD ± 31.1, range 59–210). Demographics and characteristics are demonstrated in Table 1.

## 3. Surgical Technique

Patients were placed in the beach chair position and a deltopectoral approach was used. The surgical technique has been described before [12]. In all patients, the same type of standard long-stem reverse prosthesis (Aequalis Reverse II, Memphis, TN, USA) was implanted. Implant sizes were planned preoperatively on standard radiographs. Resection of the humeral head was performed in a free-hand technique and the humeral implant was fixed with vacuum bone cement. On the glenoid side, the baseplate was fixed with two cortical and two locking screws.

## 4. Clinical Analysis

The Constant Score (CS) was used to assess functional results at the most recent FU for all patients. The score was first published in 1987 by Constant and Murley [22] and consists of four subcategories: pain (15 points maximum), activity of daily living (ADL; 20 points maximum), range of movement (ROM; 40 points maximum) and strength (25 points maximum). In sum, a total of 100 points can be reached and higher scores are interpreted as lower levels of impairment [22]. The subcategory “strength” was measured according to the method described by Constant et al. [23]: An ISOBEX dynamometer (Cursor AG, Bern, Switzerland) was used to measure the patients’ strength at 90 degrees (°) of abduction in the scapular plane with a pronated hand position and a strap applied to the level of the wrist at maximum span. The value used for the score was the maximum of three repetitions, each separated by one minute. The strength value was then converted into points between zero and 25, using a conversion table. Patients who could not achieve 90° of abduction received zero points. As strength has been identified as an age- and gender-dependent parameter, the age- and gender-adjusted CS was calculated by dividing the obtained score of the patients by the age and gender matched score of the Constant population [23].

Five patients were not able to travel and were thus contacted via telephone. In these cases, a validated German version of the CS was sent to the patients for self-assessment [24]. Patients’ satisfaction with the surgery result was assessed with a questionnaire of the institution (0–4 points; 0 points = not satisfied; 4 points = very satisfied). Diagnoses, demographics and revision rates were recorded from the patients’ medical records.

## 5. Radiographic Analysis

A total of 22 (81%) patients were available for radiographic assessment. Radiographic analysis of RSAs was performed by two surgeons who specialize in shoulder surgery. The surgeons assessed the radiographs for signs of loosening, polyethylene wear and inferior scapular notching and reached a consensus. A reduced distance between metaphysis and glenoid sphere over time was interpreted as an indirect sign of polyethylene wear and inferior scapular notching was assessed according to the classification of Sirveaux [10].

## 6. Statistics

SPSS Statistics Version 25.0.0.1 for Microsoft Windows (SPSS Inc., Chicago, IL, USA) and Microsoft Excel Version 16.54 was used for statistical analysis. Mean, standard deviation and range were calculated for continuous variables and numbers and percentages for categorical data. Non-normally distributed data were reported in median and range. Differences between pre- and postoperative data were calculated using the Wilcoxon Signed-Rank Test for non-normally distributed data. The Spearman’s Correlation Coefficient was used to calculate the statistical dependence between the rankings of two variables. Mean survival time of prostheses (with revision surgery as the end point) was assessed with the Kaplan–Meier estimator. The level of significance was set at *p* < 0.05.

## 7. Results

### 7.1. Clinical Results

At final follow-up, the mean CS improved significantly from 20.0 (range 0–41) points to 62.0 (range 21–98) points at final follow-up (*p* < 0.001). The age- and gender-adjusted Constant Score (aCS) improved from 30.0 (range 10–59) points preoperatively to 95.0 (range 33–141) points at final follow-up (*p* < 0.001). Patients with a FU over 10 years did not differ from patients with a FU under 10 years in their results of the aCS (*p* = 0.092).

External rotation improved from 0 (range from −20 to 45) degrees to 10.0 (range 0–50) degrees without statistical significance (*p* = 0.104). Forward elevation and abduction improved from 60.0 (range 10–150) degrees and 60.0 (range 20–140) degrees to 120.0 (range 30–160) degrees and 110.0 (range 0–160) degrees (*p* < 0.001; *p* < 0.001), respectively. Activity module of the CS improved from 8.0 (range 3–14) points to 18.0 (range 6–20) points and mobility improved from 8.0 (range 0–30) points to 24.0 (range 2–38) points (*p* < 0.001; *p* < 0.001). Strength improved from 0 (range 0–8) points to 5.0 (range 0–12) points (*p* < 0.001).

Pain improved from 4.5 (range 0–15) points to 15.0 (range 5–15) points on the CS Visual Analogue Scale (0–15, 0 = maximum pain) (*p* < 0.001). A total of 26 patients reported that they were satisfied (48%) or very satisfied (48%) with the procedure. One patient who underwent revision surgery with a liner exchange was less satisfied. Overall patient satisfaction was rated with 3.0 (range 2–4) points (0–4 points, very satisfied = 4 points). Patients with less pain had higher levels of satisfaction, higher scores in the aCS and reached better range of motion in forward flexion and abduction (all *p* < 0.001). Patients with a shorter operation time were found to have higher scores in the aCS (*p* = 0.013). For clinical outcomes, see Table 2.

Preoperative clinical outcomes and at final follow-up are reported in median and range for 27 patients (CS = Constant Score, aCS = age- and gender-adjusted Constant Score. The four subcategories of the CS are presented: pain (15 points maximum), activity of daily living (ADL; 20 points maximum), range of movement (ROM; 40 points maximum) and strength (25 points maximum). In sum, a total of 100 points can be reached and higher scores are interpreted as lower levels of impairment [22]. Differences between pre- and postoperative data were calculated using the Wilcoxon Signed-Rank Test for non-normally distributed data.

### 7.2. Revisions

**Figure 2:**
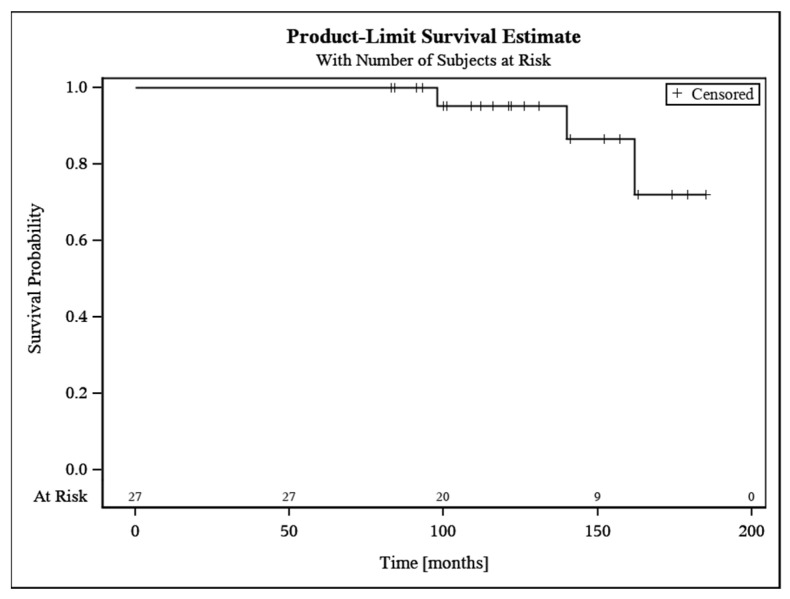
Survival analysis.

Revision surgery was necessary due to polyethylene wear in three cases (11.1%). One patient had repetitive subluxations which was found to be caused by polyethylene wear after 13 years. In two other patients, pain was the main clinical symptom. In both cases, no signs of humeral or glenoid component loosening were found intraoperatively; however, polyethylene wear was visible. All three patients were treated with liner exchange. Survivorship free of revision was 95.2% at ten years (95% CI 86.1–100). For details of revision surgery, see Table 3. For survival time analysis, see Figure 2.

Symptoms, pathology, time until revision and revision procedure among 27 RSAs.

Kaplan–Meier Estimate demonstrating survival time of 27 RSAs. Survivorship free of revision was rated with 95.2% (95% CI 86.1–100) at 10 years.

### 7.3. Radiographic Outcome

At final FU, glenoid component loosening was found in two (9.1%) cases, and humeral component loosening was found in three (13.6%) cases. At the time of clinical presentation, all patients with component loosening had little clinical impairment and opted for non-operative treatment. Polyethylene wear was observed in four (18.2%) cases. Scapular notching appeared in fifteen (68.2%) cases with three (20%) cases classified as grade 1 (according to the classification system of Sirveaux [10]), seven (46.7%) cases classified as grade 2 and five (33.3%) cases classified as grade 3. Patients with glenoid (*p* < 0.001) and humeral component loosening (*p* = 0.002) had significantly more pain.

No statistically significant impact of scapular notching on satisfaction, aCS, pain or external rotation was found (*p* > 0.05). High-grade scapular notching (grade three) was not associated with lower results in the aCS compared to notching grade one and two (*p* > 0.05). No association was found between scapular notching and glenoid component loosening (*p* > 0.05).

**Figure 3:**
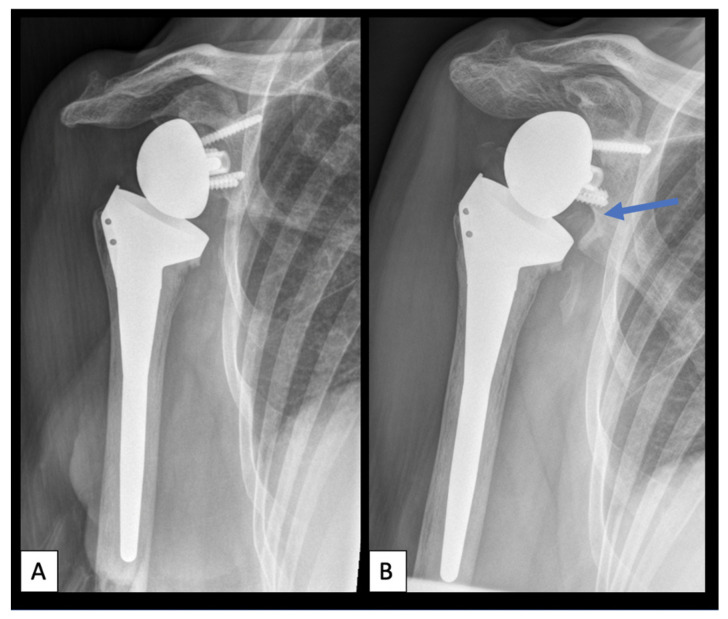
Scapular notching. (**A**): Anteroposterior radiograph of a right shoulder one-year postoperatively. (**B**): Anteroposterior radiograph of a right shoulder eight years postoperatively with scapular notching grade three according to the classification of Sirveaux [10]. Blue arrow demonstrating the area of scapular notching at the inferior glenoid.

For radiographic outcomes, see Table 4. Figure 3 demonstrates an example of scapular notching eight years postoperatively.

Component loosening, polyethylene wear and scapular notching rates among 22 RSAs.

## 8. Discussion

The most important finding of this study was a significant improvement of the aCS from 30.0 (range 10–59) points preoperatively to 95.0 (range 33–141) points at a mean clinical FU of 10.6 years (*p* < 0.001). Further, all subcategories of the CS (activity, mobility, strength and pain) were improved at final FU (*p* < 0.001). Our results demonstrate that 96% of the patients were satisfied or very satisfied with the procedure and that pain had significantly improved at final FU (*p* < 0.001).

Our results are comparable to the long-term results by Favard et al. [12], who demonstrated a significantly improved aCS for 148 patients with CTA, massive rotator cuff tears and primary OA after a mean FU of 7.5 years. Another study on long-term outcome data was conducted by Ek et al. [25], who found a significantly improved aCS of 40 shoulders with painful pseudoparesis secondary to massive irreparable rotator cuff tears with and without OA after a mean FU of 7.8 years. Interestingly, Favard et al. [12] demonstrated that patients with a minimum FU of seven and nine years had lower aCS scores than those with less than five years of FU. In our study, patients with a FU over 10 years did not differ from patients with a FU under 10 years in their results of the aCS (*p* > 0.05). Throughout the literature, other groups have shown similar good results for patients with CTA, rotator cuff deficiency and OA in the mid- to long-term FU [10,26,27,28,29,30,31] (for an overview of recent literature on clinical outcome data, see Table 5).

Functional improvements were further found for range of motion. Forward elevation and abduction improved from 60.0 (range 10–150) degrees and 60.0 (range 20–140) degrees to 120 (range 0–160) degrees and 110 (range 0–160) degrees (both *p* < 0.001) while external rotation was improved but without statistical significance. It has been previously shown that treatment with RSA can restore forward elevation to a relevant extent [10,11]; however, only limited improvements in active external rotation must be expected [15,20]. Boileau et al. [15] demonstrated that an intact teres minor muscle has a direct influence on the ability of postoperative active external rotation, but the status of this specific muscle was not concisely documented in this study. In recent years, design modifications such as a reduction in the neck–shaft angle, the use of more lateralized designs and larger glenospheres as well as new techniques such as additional tendon transfers were introduced to improve active range of motion [20,34,35].

The second important finding of this study was that scapular notching appeared in more than half of the cases (68.2%) but no impact of scapular notching on satisfaction, aCS, pain or external rotation was found. Additionally, high-grade scapular notching (grade three) was not associated with lower results in the aCS compared to notching grade one and two. Other study groups have found similar notching rates such as Sirveaux et al. [10] (63.6%), Boileau et al. [26] (67%) and Al-Hadithy et al. [28] (68%). While one group found decreased CS in cases of higher notching grades [10], others did not find a statistical association [26,28]. While the true impact of notching on clinical outcome remains a matter of controversy, larger sample sizes as demonstrated by Mollon et al. [36] are crucial to gain further information. This group evaluated 476 RSAs after CTA, cuff tear deficiency and OA at a mean FU of 38 months and found scapular notching related to lower results in the CS and longer clinical FU.

The third important finding of the study was a revision rate of 11.1%, which is in line with reported revision rates after the implantation of RSA for CTA, rotator cuff deficiency and OA in the literature (between 2.4% [28] and 27.5% [25]). With a survivorship of 95.2% at ten years, our study confirms the 10 year survival rates (survivorship free of revision) reported by Favard et al. (89%) [12] and Ek et al. (76%) [25]. Of note, in our study, glenoid component loosening was found in two (9.1%) cases and humeral component loosening was found in three cases (13.6%). All patients were informed about the radiographic observation but at the time of clinical presentation, all patients with component loosening had little to no clinical impairment and opted for non-operative treatment.

Instability, component loosening, soft tissue defects, periprosthetic fractures, acromial stress fractures and infection are possible causes of failure [10,37,38]. In this study, one patient experienced repetitive subluxations as a sign of instability and was revised to a larger liner 13 years postoperatively. Instability has been reported as a common cause of revision after RSA and known risk factors are component malpositioning, inadequate soft tissue tensioning, prosthesis design, surgical approach, high body mass index, male sex and subscapularis deficiency [37,39]. For this patient, polyethylene wear was suspected to be the mechanical reason for instability but also soft tissue degeneration may have caused the long-term complication.

While glenoid component loosening has been identified as one of the most common reasons for revision surgery [13], humeral component loosening is considered a rather rare complication after RSA [37]. However, revision of a well-fixed stem can be technically challenging and often requires corticotomy and bone grafting [37]. As a consequence, one new trend in the field of RSA is the shortening of the humeral component to prevent humeral bone stock and facilitate revision surgery [40]. Promising clinical results can be achieved with this new technique; however, there is a paucity of long-term data [41]. Potential risk factors for subsequent humeral loosening, such as subsidence and the presence of high bone adaptions, have been identified in the short- and midterm follow-up [21,41,42]. So far, the new implants are predominantly used by specialized shoulder surgeons and it remains unclear whether there is superiority over the traditional long-stem Grammont-style prosthesis. In a study by Merolla et al. [43], a reverse short humeral stem was directly compared to a Grammont-style RSA and no difference regarding function and complication rates was found in the short-term FU. The reported results demonstrate that standard cemented RSA can still be recommended and long-term controlled outcome studies in the future are needed for further conclusions on designs.

## 9. Limitations

Our results are limited by the small sample size and the lack of a control group. Data were analyzed retrospectively. No information about possible revisions can be provided for patients with loss of follow-up; therefore, the true revision rate could be higher than the presented number.

## 10. Conclusions

RSA with a cemented standard long stem leads to improvement in forward elevation, abduction and pain after a mean FU of 10 years. However, external rotation does not improve with this prosthetic design. Moreover, scapular notching is observed in the majority of cases, and revision rates (11.1%) as well as humeral loosening rates (13.6%) remain a concern.

## Figures and Tables

**Table 1:** Demographics and characteristics of the study cohort. Age * = age at the time of implantation; SD = standard deviation; FU = follow-up.

Demographic Variable	Value (*n* = 27)
Age *, years, mean (SD; range)	72.6 (5.4; 61–84)
Gender, female/male; *n* (%)	23 (85)/4 (15)
Primary osteoarthritis, *n* (%)	2 (7.4)
Cuff tear arthropathy, *n* (%)	25 (92.6)
Clinical FU, months, mean (SD; range)	127.6 (33.7; 83–185)
Radiographic FU, months, mean (SD; range)	126.4 (34.3; 83–185)
Operation time, minutes, mean (SD; range)	98 (31.1; 59–210)

**Table 2:** Clinical outcomes. CS= Constant Score; aCS= age- and gender-adjusted Constant Score.

Variable	Preoperative	Follow-Up	*p*-Value
CS, median (range)	20.0 (0–41)	62.0 (21–98)	*p* < 0.001
aCS, median (range)	30.0 (10–59)	95.0 (33–141)	*p* < 0.001
Activity, median (range)	8.0 (3–14)	18.0 (6–20)	*p* < 0.001
Mobility, median (range)	8.0 (0–30)	24.0 (2–38)	*p* < 0.001
Strength, median (range)	0 (0–8)	5.0 (0–12)	*p* < 0.001
Pain, median (range)	4.5 (0–15)	15.0 (5–15)	*p* < 0.001
Forward elevation, degree, median (range)	60.0 (0–150)	120 (30–160)	*p* < 0.001
External rotation, degree, median (range)	0 (−20–45)	10.0 (0–50)	*p* = 0.104
Abduction, degree, median (range)	60.0 (20–140)	110.0 (0–160)	*p* < 0.001

**Table 3:** Revisions.

Symptoms and Pathology	Time until Revision	Revision Surgery
Instability; polyethylene wear	13 years	Exchange of liner
Pain; polyethylene wear	9 years	Exchange of liner
Pain; polyethylene wear	7 years	Exchange of liner

**Table 4:** Radiographic outcomes.

Radiographic Observation	Absolute Numbers and Percentage
Stem loosening, *n* (%)	3 (13.6)
Glenoid loosening, *n* (%)	2 (9.1)
Polyethylene wear, *n* (%)	4 (18.2)
Scapular notching, *n* (%)	
Grade 1	3 (20%)
Grade 2	7 (46.7%)
Grade 3	5 (33.3%)

**Table 5:** Overview of recent literature on clinical outcome data. CTA = cuff tear arthropathy; RCD = rotator cuff deficiency; OA = primary osteoarthritis.

Author and Year of Publication	N	Indication	Mean FU (Months)	CS Pre/Post	aCS Pre/Post	Forward Elevation Pre/Post	Glenoid Loosening Rate (%)	Stem Loosening Rate (%)	Revision Rate (%)	Scapular Notching Rate (%)
Jacobs et al., 2001 [14]	7	CTA	16	18/57	NA	NA	0	NA	NA	NA
Sirveaux et al., 2003 [10]	80	CTA	44	23/66	NA	73/138	6	NA	5	63.6
Vanhove et al., 2004 [32]	14	CTA	31	NA/60	NA	NA	NA	NA	NA	50
Seebauer et al., 2005 [33]	57	CTA, RCD	18	NA/67	NA/94	NA/145	0	NA	NA	24
Boileau et al., 2009 [26]	46	RCD	50	25/56	36/79	82/123	0	2.2	4.3	67
Favard et al., 2011 [12]	148	CTA, OA, RCD	90	24/62	33/85	69/129	NA	NA	NA	35 (grade 3 and 4)
Mizuno et al., 2013 [31]	27	OA	54	31/76	NA	89/152	3.7	0	3.7	37
Ek et al., 2013 [25]	40	RCD	93		34/74	72/119	7.5	0	27.5	56
Raiss et al., 2014 [27]	13	CTA	42	26/67	NA	70/130	0	0	8	38
Al-Hadithy et al., 2014 [28]	41	RCD	60	24/60	34/71	55/108	0	0	2.4	68
Gruber et al., 2017 [29]	39	CTA, RCD, OA	68	NA	39/71	NA/39	NA	NA	7.7	64
Boileau et al., 2020 [30]	143	RCD	75	40/93	NA	84/137	0	0	4	56

Studies reporting on clinical outcome data with mean FU between 16 and 93 months.
